# A machine learning-based risk warning platform for potentially inappropriate prescriptions for elderly patients with cardiovascular disease

**DOI:** 10.3389/fphar.2022.804566

**Published:** 2022-08-11

**Authors:** Wu Xingwei, Chang Huan, Li Mengting, Qin Lv, Zhang Jiaying, Long Enwu, Zhu Jiuqun, Tong Rongsheng

**Affiliations:** ^1^ Personalized Drug Therapy Key Laboratory of Sichuan Province, Department of Pharmacy, Sichuan Provincial People’s Hospital, School of Medicine, University of Electronic Science and Technology of China, Chengdu, Sichuan, China; ^2^ Chinese Academy of Sciences Sichuan Translational Medicine Research Hospital, Chengdu, Sichuan, China; ^3^ Department of Pulmonary and Critical Care Medicine, Sichuan Academy of Medical Sciences and Sichuan Provincial People’s Hospital, Chengdu, China; ^4^ Department of Western Pharmacy, Chengdu First People’s Hospital, Chengdu, China

**Keywords:** cardiovascular diseases, potentially inappropriate prescribing, potentially inappropriate medications, potential prescribing omissions, machine learning, predictive models

## Abstract

Potentially inappropriate prescribing (PIP), including potentially inappropriate medications (PIMs) and potential prescribing omissions (PPOs), is a major risk factor for adverse drug reactions (ADRs). Establishing a risk warning model for PIP to screen high-risk patients and implementing targeted interventions would significantly reduce the occurrence of PIP and adverse drug events. Elderly patients with cardiovascular disease hospitalized at the Sichuan Provincial People’s Hospital were included in the study. Information about PIP, PIM, and PPO was obtained by reviewing patient prescriptions according to the STOPP/START criteria (2nd edition). Data were divided into a training set and test set at a ratio of 8:2. Five sampling methods, three feature screening methods, and eighteen machine learning algorithms were used to handle data and establish risk warning models. A 10-fold cross-validation method was employed for internal validation in the training set, and the bootstrap method was used for external validation in the test set. The performances were assessed by area under the receiver operating characteristic curve (AUC), and the risk warning platform was developed based on the best models. The contributions of features were interpreted using SHapley Additive ExPlanation (SHAP). A total of 404 patients were included in the study (318 [78.7%] with PIP; 112 [27.7%] with PIM; and 273 [67.6%] with PPO). After data sampling and feature selection, 15 datasets were obtained and 270 risk warning models were built based on them to predict PIP, PPO, and PIM, respectively. External validation showed that the AUCs of the best model for PIP, PPO, and PIM were 0.8341, 0.7007, and 0.7061, respectively. The results suggested that angina, number of medications, number of diseases, and age were the key factors in the PIP risk warning model. The risk warning platform was established to predict PIP, PIM, and PPO, which has acceptable accuracy, prediction performance, and potential clinical application perspective.

## 1 Introduction

With the rapid aging of the global population, countries around the world are currently facing a serious problem of an aging population and the health problems of the elderly. It is estimated that by 2050, the number of people aged over 60 years will reach 2.1 billion worldwide (Phillips, 2017), and the proportion will be more than 20% (Biritwum et al., 2013). The elderly have poor physical function and often suffer from comorbidities (Vermunt et al., 2017). Costs related to comorbidities are a significant economic burden to patients and healthcare systems (King and Giedrimiene, 2021). During recent years, there has been a growing interest in the study of disease associations in aging patients. Accumulated evidence has suggested that cardiovascular diseases are the most common comorbid condition in older people with multimorbidities (Manckoundia et al., 2020). In addition, cardiovascular diseases are considered a leading cause of death, and the rate reached 10%–30% in the LifeLines Cohort Study (Van der Ende et al., 2017). Importantly, this becomes even more pronounced in the elderly population (Hamilton-Craig et al., 2015; Valdés et al., 2018).

Moreover, elderly patients usually have several comorbidities that leads to polypharmacy, increasing the risk of potentially inappropriate prescribing (PIP) (Corsonello et al., 2010; Gallagher et al., 2011; D'Cruz et al., 2012; Chen et al., 2014; Hyttinen et al., 2019b), especially leading to an increase in adverse drug reactions (ADRs) (Maaroufi et al., 2021). Additionally, PIP includes potentially inappropriate medications (PIM) and potential prescribing omissions (PPO), which is a key factor influencing the occurrence of ADR in elderly patients (O’Mahony and Gallagher, 2008). PIM is very common in elderly patients with cardiovascular disease (Sheikh-Taha and Dimassi, 2017; Maaroufi et al., 2021). 98.2% of elderly patients, who were admitted to medical or cardiovascular ICUs in a large tertiary teaching hospital in Brazil, had at least one PIM (Galli et al., 2016). According to a multicenter, prospective cohort study that recruited 1,280 patients (median age of 82 years) in England, PIM contributed to ADR in 12% of elderly patients (Parekh et al., 2019).

Currently, there are various criteria for assessing PIP (Petrovic et al., 2016; Lopez-Rodriguez et al., 2020); for instance, the Beers criteria developed in the United States (By the 2019 American Geriatrics Society Beers Criteria^®^ Update Expert Panel, 2019) and the STOPP/START criteria developed in Ireland (O’Mahony et al., 2015). Although these criteria are currently in wide use for post-event evaluation, there are some shortcomings, such as the inability to provide advance warning of the risk of PIP in elderly patients. Through early warning of PIP, physicians or pharmacists will be able to identify patients at risk of PIP and adopt individualized interventions to reduce the risk of ADR.

Some studies have shown that PIP in elderly patients can be identified using the frailty index (Cullinan et al., 2016) and the new Croatian tool (Matanović and Vlahović-Palčevski, 2014). However, these approaches were not convenient enough, as they would require a lot of time and effort. In recent years, with the rise of artificial intelligence, machine learning algorithms have been increasingly applied to develop predictive models (Badet et al., 2021; Fralick et al., 2021; Hossain et al., 2021; Lin et al., 2021; Mišić et al., 2021; Pinaire et al., 2021). Multiple studies reported that machine learning algorithms could predict severe hypoglycemia in hospitals, identify genetic risk factors for the progression and survival of colorectal cancer, etc. Patel et al. used machine learning algorithms to develop predictive models to identify predictors of inappropriate use of non-steroidal anti-inflammatory drugs (NSAIDs) of PIP in elderly patients with osteoarthritis (Patel et al., 2020).

However, the following problems remain to be resolved: 1) Fewer data pre-processing methods are used. Our previous study (Wu et al., 2020) has demonstrated that different data pre-processing methods (*p* < 0.05) are important in choosing an optimal data pre-processing method. 2) Fewer machine learning algorithms are used. Our previous study (Wu et al., 2020) used 14 machine learning algorithms, and the results showed the variability between different machine learning algorithms. Each machine learning algorithm applies in different conditions. At most two machine learning algorithms in the above studies were used, which was not sufficient. 3) Fewer platforms for risk prediction. Risk warning platforms can output the risk of PIP in elderly patients with cardiovascular disease, which might alert physicians or pharmacists to review the medicines.

Thus, the present study analyzed the information on PIP, PIM. and PPO of cardiovascular disease in elderly patients, and established a prediction platform using multiple machine learning algorithms to predict the risk of PIP, PIM, and PPO in elderly patients with cardiovascular disease.

## 2 Materials and methods

### 2.1 Data sources

**FIGURE 1:**
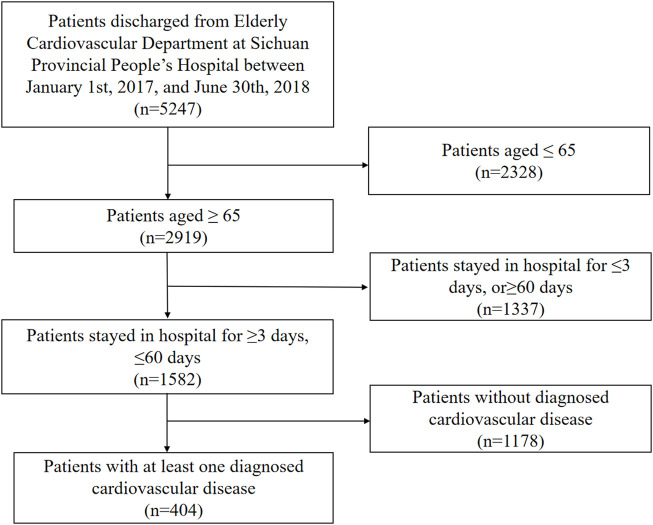
Flowchart of patient selection.

Participants who were discharged from the Department of Geriatric Cardiology at Sichuan Provincial People’s Hospital from January 2017 to June 2018 were included in this study. Their clinical information, including prescription information, medical record information, and results of laboratory tests, were collected from the electronic medical record. The following inclusion criteria were used for the selection of the study participants: 1) age ≥65 years; 2) the duration of hospitalization between 3 and 60 days; and 3) diagnosed with at least one cardiovascular disease (hypertension, myocardial infarction, angina pectoris, hyperlipidemia, peripheral vascular disease, and indication for antithrombotic therapy, which was determined by cardiovascular physicians). The patient selection flowchart is shown in Figure 1.

The STOPP/START criteria (version 2) for the cardiovascular system and antiplatelet/anticoagulant drugs were used to identify PIP prescriptions in elderly patients, including 24 PIM criteria (13 for cardiovascular system and 11 for antiplatelet/anticoagulant drugs) and 8 PPO criteria for the cardiovascular system. Each electronic medical record was independently reviewed by three pharmacists, Wu Xingwei, Zhang Jiaying, and Xiong Huan, who had received training from the chief pharmacists (Tong Rongsheng and Long Enwu) to ensure the accuracy of the results. All disagreements were resolved by consulting the chief physician of internal medicine.

The patient’s ID number, name, home address, and telephone number were anonymous during the data acquisition for ethical reasons. As this is a retrospective study without intervention, the ethics committee considered it unnecessary to obtain informed consent from patients. All variables were coded (X1, X2, … , Xn) to allow blinded analysis of patient data.

### 2.2 Data pre-processing

#### 2.2.1 Data pre-screening

Data pre-screening included three processes: 1) deleting columns with more than 90% missing data; 2) deleting columns with a single value occupying more than 90%; and 3) deleting columns with coefficient of variation less than 0.01. Variables meeting one of the abovementioned conditions would be considered less informative.

#### 2.2.2 Data sampling

To minimize the adverse impact of data imbalance on prediction performance, the following data sampling methods were used: 1) no sampling; 2) random upsampling, which duplicates minority class samples to create additional minority class samples; 3) random undersampling, which randomly selects samples from the initial dataset to create a new smaller dataset; 4) synthetic minority oversampling technique (SMOTE), which achieves upsampling by linear interpolation between a small number of class samples and their nearest neighbors; and 5) borderline SMOTE upsampling, which improves the SMOTE method by upsampling only the border samples of a small number of classes, thus improving the class distribution of the samples.

#### 2.2.3 Feature screening

Three feature selection methods were used for feature screening: 1) no screening; 2) the Lasso screening method which evaluates the importance of variables and output the results by introducing a penalization parameter penalizing and discarding unimportant variables (variables with coefficients near zero); and 3) the Boruta screening method which is a feature selection algorithm to identify the minimal set of relevant variables.

### 2.3 Model development

Fifteen datasets were generated by five data sampling methods and three feature screening methods, and 18 machine learning algorithms were used on each dataset, respectively, to develop a total of 270 models. Machine learning algorithms in this study included logistic regression, decision tree, Gaussian naive bayes, Bernoulli naive bayes, multinomial naive bayes, passive aggressive, AdaBoost, bagging, gradient boosting, eXtreme gradient boosting (XGBoost), K-nearest neighbor (KNN), linear discriminant analysis (LDA), quadratic discriminant analysis (QDA), random forest, stochastic gradient descent (SGD), support vector machine (SVM), extra tree, and ensemble learning (Wu et al., 2020). These abovementioned algorithms were commonly used and were suitable for binary classification. In comparison to single classification algorithms, ensemble algorithms always prove to be more effective and stable in prediction models.

The whole process of model development could be described as follows:

(1) The data set was divided into a training set and a test set in a ratio of 8:2 (according to our sample size, a ratio of 8:2 would be more suitable than 9:1 or 7:3)
(2) Models were trained in the training set so that the loss function was minimized. Internal validation was performed by the ten-fold cross-validation method.
(3) Test set data were passed into the trained model for assessing model prediction performance. Bootstrapping was employed for external validation.
(4) The model with the best performance was selected.

### 2.4 Model evaluation

**FIGURE 2:**
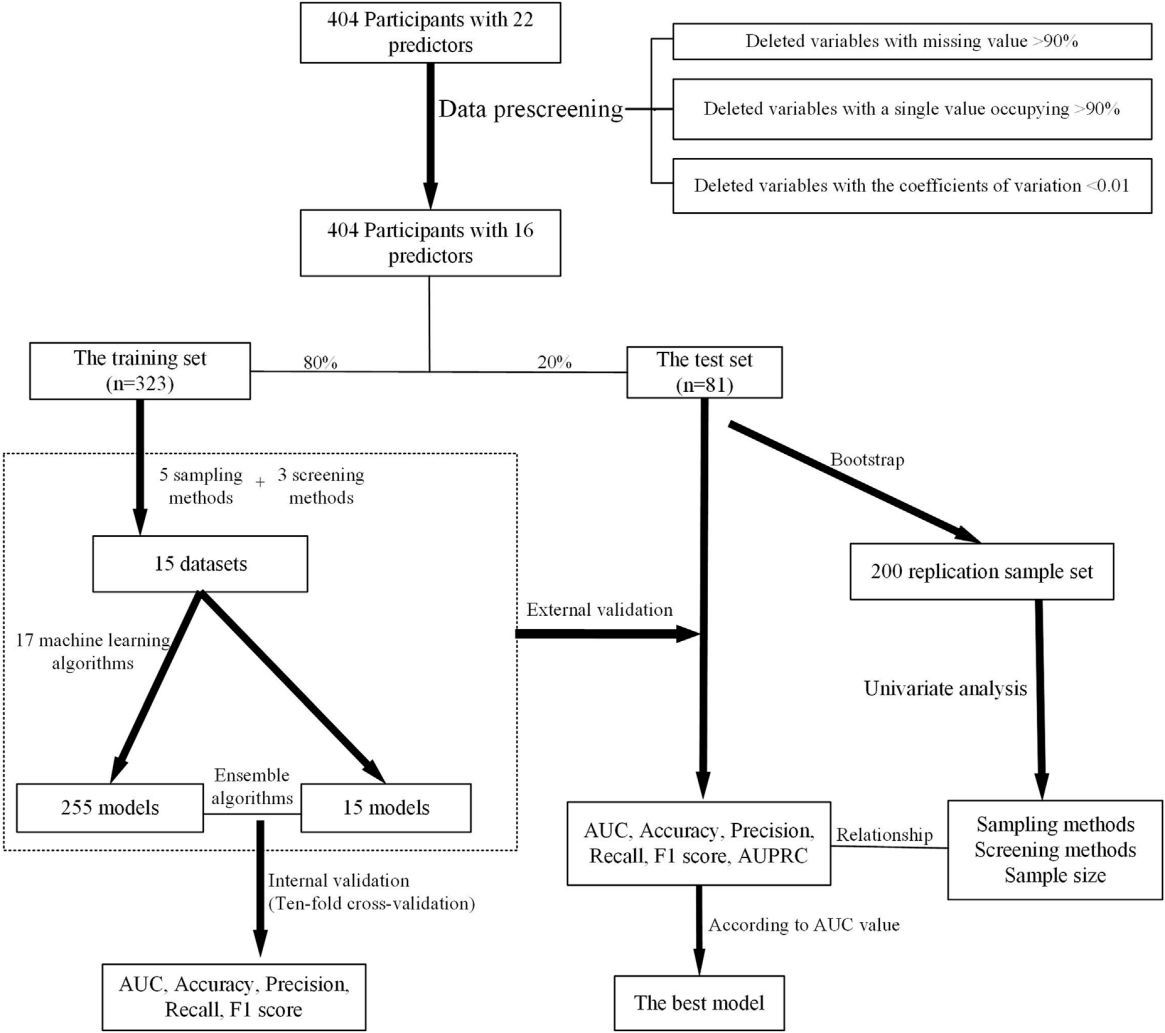
Overview of the modeling method.

The area under the receiver operating characteristic curve (AUC), accuracy, precision, recall, and F1 score were adopted as quantitative metrics to evaluate the performance of models, and the candidate model achieving the best performance was selected as the optimal prediction model. The contribution of each variable to the predictive model was estimated with SHapley Additive exPlanation (SHAP). The modeling process is shown in Figure 2.

A total of 270 prediction models were developed based on different sampling methods and feature screening methods. On the test set, the model with the highest AUC value was selected and used to establish the prediction platform for PIP, PIM, and PPO.

### 2.5 Sample size validation

To train the best model, the bootstrap method was used to randomly select 10%, 20%, 30%,......100% of the resampling data from the training set, and the test set was used to test the predictive ability of the model. The AUC values of the best models of PIP, PPO, and PIM were estimated. The above process was repeated 100 times, and the results were plotted on a line graph. We judged the contribution of the sample size to improve the prediction performance of models according to the inflection point change of the line graph.

### 2.6 Statistical analysis

Categorical data were described by frequency (percent), and continuous data were statistically described by mean and standard deviation (Mean ± SD). Analysis of variance (ANOVA) and rank sum test were used for univariate analysis.

Statistical analysis was performed using stats in Python 3.8, and model development was implemented using sklearn in Python 3.8. The front-end of the PIP prediction platform was written in JavaScript, and the back-end was written in Python 3.8.

## 3 Results

A total of 404 elderly patients with cardiovascular diseases were eligible for this study. The mean age of the patients was 79.1 years, and 59.9% were male. Participants identified as PIP, PIM, and PPO were 318 (78.7%), 112 (27.7%), and 273 (67.6%), respectively. The most frequent PIPs were antiplatelet agents simultaneously used with vitamin K antagonists, direct thrombin inhibitors, or factor inhibitors in patients (37 instances, accounting for 21.5% of total PIPs). Table 1 shows detailed patient demographic information and clinical information as the independent variables, with PIP, PIM, and PPO as the dependent variables.

**TABLE 1:** Information of PPO, PIP, PIM, and characteristics in the participants.

No.	Variable	Parameter	Value (N = 404)
	PIP	No	86 (21.3%)
		Yes	318 (78.7%)
	PPO	No	131 (32.4%)
		Yes	273 (67.6%)
	PIM	No	292 (72.3%)
		Yes	112 (27.7%)
X1	Gender	Female	242 (59.9%)
		Male	162 (40.1%)
X2	Age (years)		79.1 ± 8.18
X3	Duration of hospital stay (days)		19.5 ± 9.96
X4	Number of diseases		6.3 ± 2.45

X5	Number of medications		16.2 ± 9.74

X6	Hypertension	No	95 (23.5%)
		Yes	309 (76.5%)
X7	Cerebrovascular disease	No	215 (53.2%)
		Yes	189 (46.8%)
X8	Myocardial infarction	No	390 (96.5%)
		Yes	14 (3.5%)
X9	Angina	No	279 (69.1%)
		Yes	125 (30.9%)
X10	Heart failure	No	235 (58.2%)
		Yes	169 (41.8%)
X11	Heart block	No	370 (91.6%)
		Yes	34 (8.4%)
X12	Atrial fibrillation	No	336 (83.2%)
		Yes	68 (16.8%)

X13	Atherosclerosis	No	93 (23.0%)
		Yes	311 (77.0%)

X14	Hyperlipidemia	No	342 (84.7%)
		Yes	62 (15.3%)
X15	Diabetes	No	280 (69.3%)
		Yes	124 (30.7%)
X16	Venous thromboembolism	No	395 (97.8%)
		Yes	9 (2.2%)
X17	History of gout	No	392 (97.0%)
		Yes	12 (3.0%)
X18	Renal failure	No	367 (90.8%)
		Yes	37 (9.2%)
X19	Peptic ulcer or alimentary tract hemorrhage	No	352 (87.1%)
		Yes	52 (12.9%)
X20	History of cardiovascular disease	No	45 (11.1%)
		Yes	359 (88.9%)
X21	Anticoagulant therapy	No	30 (7.4%)
		Yes	374 (92.6%)
X22	Antithrombotic therapy	No	119 (29.5%)
		Yes	285 (70.5%)

### 3.1 Data pre-processing

#### 3.1.1 Data pre-screening

After removing columns that met the deleting criteria, 16 variables were retained and 6 variables were deleted (X8 myocardial infarction, X11 heart block, X16 venous thromboembolism, X17 history of gout, X18 renal failure, and X21 anticoagulant therapy).

#### 3.1.2 Feature screening

After data pre-screening and data sampling, the variables were screened using the Lasso method and the Boruta method, as shown in Supplementary Figure S1. The results showed that the five most important variables in the PIP model were angina, atherosclerosis, heart failure, diabetes, and number of medications (Supplementary Figure S1A). In the PPO model, the five most important variables were number of medications, angina, atherosclerosis, and history of cardiovascular diseases (Supplementary Figure S1B). The most important variables in the PIM model were number of medications, number of diseases, duration of hospitalization (days), age, and heart failure (Supplementary Figure S1C).

### 3.2 Model validation

#### 3.2.1 Internal validation

Internal validation was performed using the 10-fold cross-validation method. Fifteen datasets were created using five data sampling methods and three feature screening methods. Two hundred and fifty-five models for predicting PIP, PPO, and PIM respectively, were built using 18 machine learning algorithms. Different data sampling methods and different machine learning algorithms in the PIP model were significantly affected by the prediction performance of the PIP model (*p* < 0.0001). Details are listed in Supplementary Table S1.

As shown in Supplementary Table S2, different data sampling methods and different machine learning algorithms showed significant differences in the prediction performance of the PPO model (*p* < 0.0001), but the different feature screening methods were not significant (*p* > 0.05).

The results of the PIM prediction model were similar to those of the PPO prediction model. Significant differences between different data sampling methods and machine learning algorithms on the prediction performance of the PIM model are shown in Supplementary Table S3.

#### 3.2.2 External validation

Applying 18 machine learning algorithms, 270 machine learning models were developed for each output. External validation of the models was performed by bootstrapping 200 samples in the test set. Different data sampling methods, different feature screening methods, and different machine learning algorithms in the PIP models had a significant effect (*p* < 0.0001) on prediction performance (list in Supplementary Table S1).

As presented in Supplementary Tables S1, S3, the results of the external validation of the PIM and PPO models were consistent with the PIP models. Data sampling methods, feature screening methods, and machine learning algorithms showed statistically significant differences in the prediction performance of the PIM and the PPO model.

#### 3.2.3 Variable importance

The data from 200 bootstrapping samples were entered in the PIP, PIM, and PPO models. The contribution of each variable to the prediction performance in the different models is shown in Supplementary Figure S2 by the averaged AUC value when the variable was included in the prediction model. The five most important variables in the PIP model were cerebrovascular disease, history of cardiovascular disease, number of medications, duration of hospitalization (days), and age, while diabetes, gastrointestinal bleeding, hypertension, and angina were the least important (Supplementary Figure S2A). In the PPO model, the five most important variables were diabetes, hyperlipidemia, heart failure, duration of hospitalization (days), and gastrointestinal bleeding, while the five least important variables were hypertension, cerebrovascular disease, antithrombotic therapy, and atrial fibrillation (Supplementary Figure S2B). The most important variable in the PIM model were diabetes, antithrombotic therapy, duration of hospitalization (days), age, and hypertension, while gastrointestinal bleeding, hyperlipidemia, history of cardiovascular disease, and atrial fibrillation were unimportant (Supplementary Figure S2C).

### 3.3 Model selection

#### 3.3.1 Model evaluation

**FIGURE 3:**
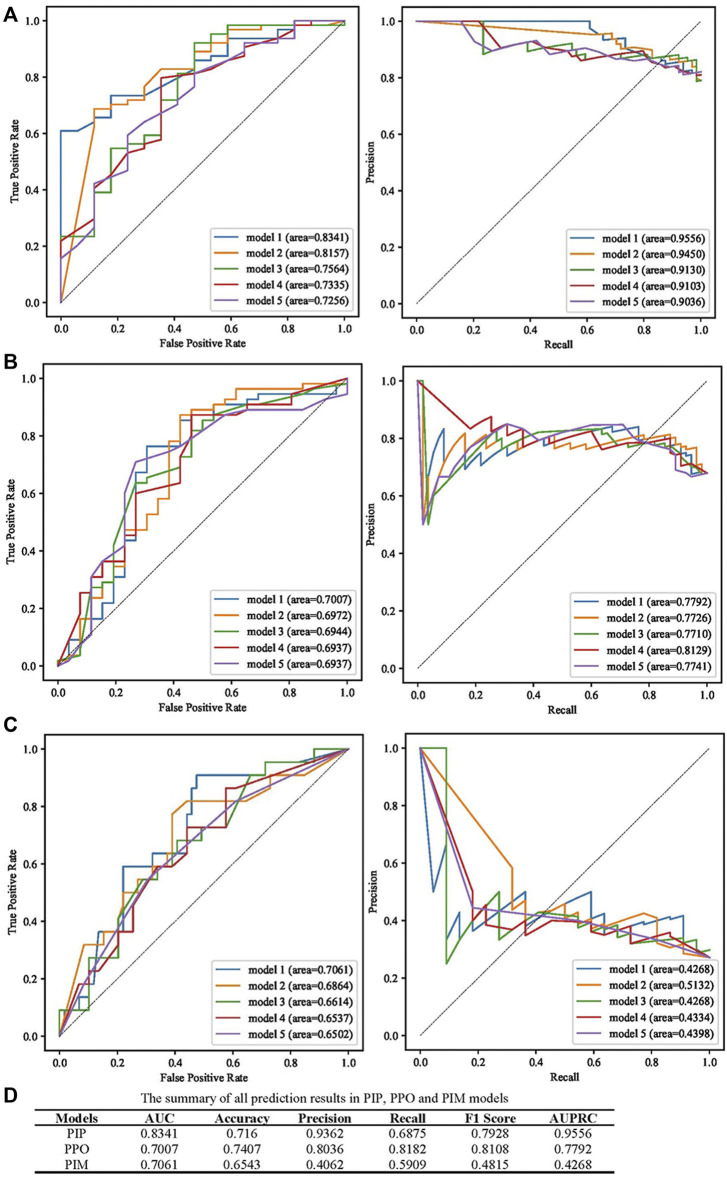
Summary of the performance of PIP, PPO, and PIM model. **(A)** The results of AUC and AUPRC in the best five PIP model. **(B)** The results of AUC and AUPRC in the best five PPO model. **(C)** The results of AUC and AUPRC in the best five PIM model. **(D)** The summary of AUC, accuracy, precision, recall, F1 score, AUPRC in the best PIP, PPO, and PIM model.

AUC, accuracy, precision, recall, F1 score, and the area under the precision-recall curve (AUPRC) were used to evaluate the predictive performance of models, and the best models according to the AUC value are presented in Figure 3. The prediction performance of the PIP model achieved an AUC of 0.8341 and an AUPRC of 0.9556 (Figure 3A). As presented in Figure 3B, the best performing PPO model had the highest AUC (0.7007) and AUPRC (0.7992). The best prediction performance of the PIM model provided an AUC of 0.7061 and an AUPRC of 0.4268 (Figure 3C). The best predictive performance metrics of PIP, PIM, and PPO are presented in Figure 3D.

#### 3.3.2 SHapley additive explanation evaluation

**FIGURE 4:**
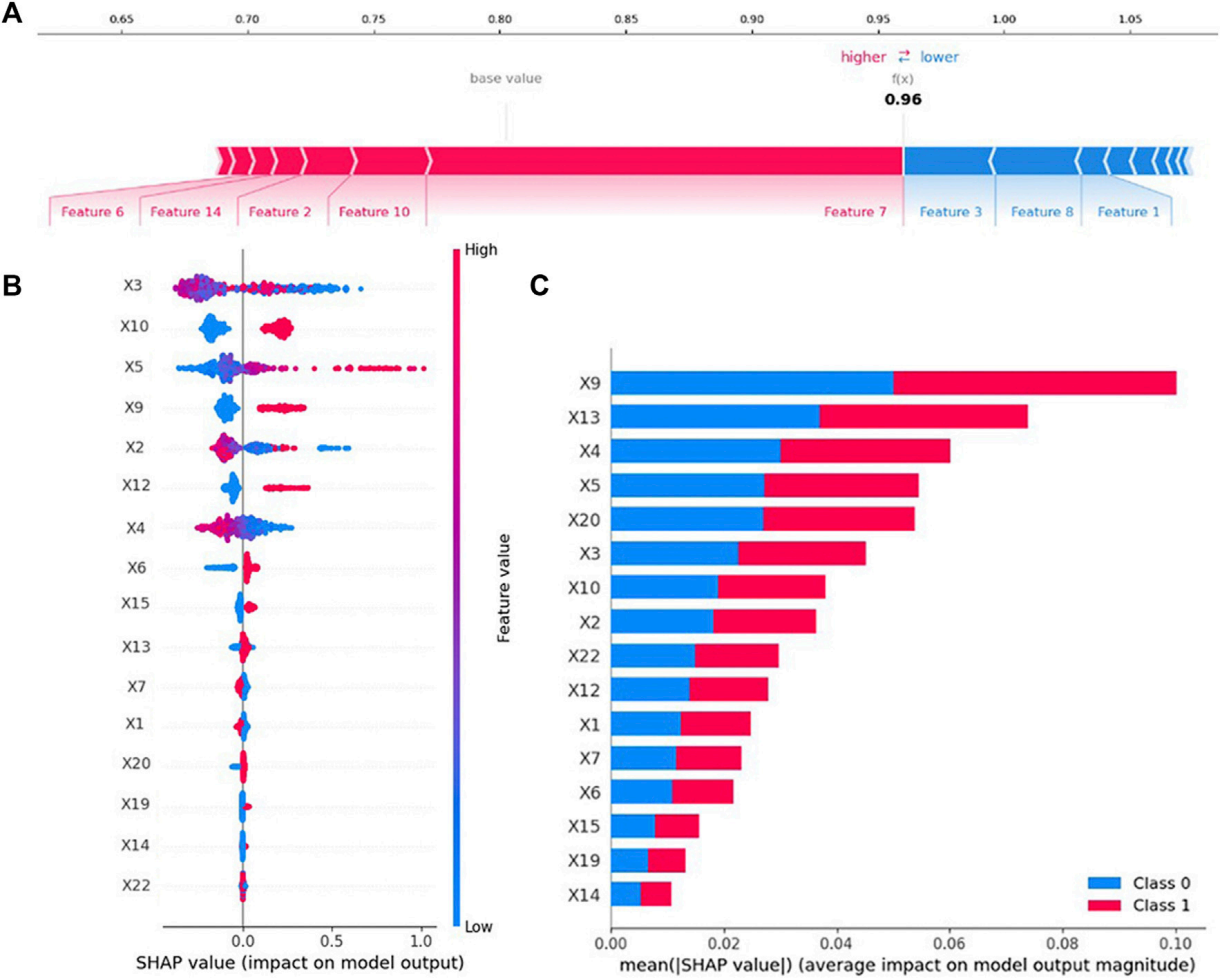
Variable contribution to the PIP model by SHAP Value. **(A)** Contribution of each feature value in one sample. **(B)** Summary of SHAP value of each variable. **(C)** Absolute average of SHAP value of each variable.

SHAP can interpret the output of any machine learning model. The contribution of variables in the PIP model is explained by SHAP, and the results are shown in Figure 4. As illustrated in Figure 4A, SHAP estimated the contribution of each feature value in each sample to the prediction. Cerebrovascular disease, heart failure, age, hyperlipidemia, and hypertension provided a positive contribution to the SHAP value, while duration of hospital stay (days), myocardial infarction, and gender provided a negative contribution. Cerebrovascular disease was the most important variable.

As presented in Figure 4B, the SHAP value of each feature in each sample was calculated and plotted. Variables were ranked in descending order by summarizing the SHAP values of each sample. For example, the higher the values of duration of hospital stay (days), lower the value of SHAP.

The mean of the absolute value of the SHAP value of each variable, which was regarded as of feature importance, was plotted as shown in Figure 4C. The top five most important variables in the PIP model were angina, atherosclerosis, number of diseases, number of medications, and history of cardiovascular disease.

### 3.4 Sample size validation

The adequacy of the sample size was verified using the resampling bootstrapping method, and the results are plotted in Supplementary Figure S3. In the PIP model, the AUC gradually increased and the dispersion of the AUC value decreased as the percentage of sample size increased. When the sample size reached 70%, the curve flattened. The results indicated that the performance of the PIP model might be affected when expanding the sample size (Supplementary Figure S3A). In both the PPO model and the PIM model, both the curves showed an upward trend. These results indicate that the performance of the PPO and PIM models might be improved even further with the addition of samples (Supplementary Figures S3B,C).

### 3.5 Development prediction platform

**FIGURE 5:**
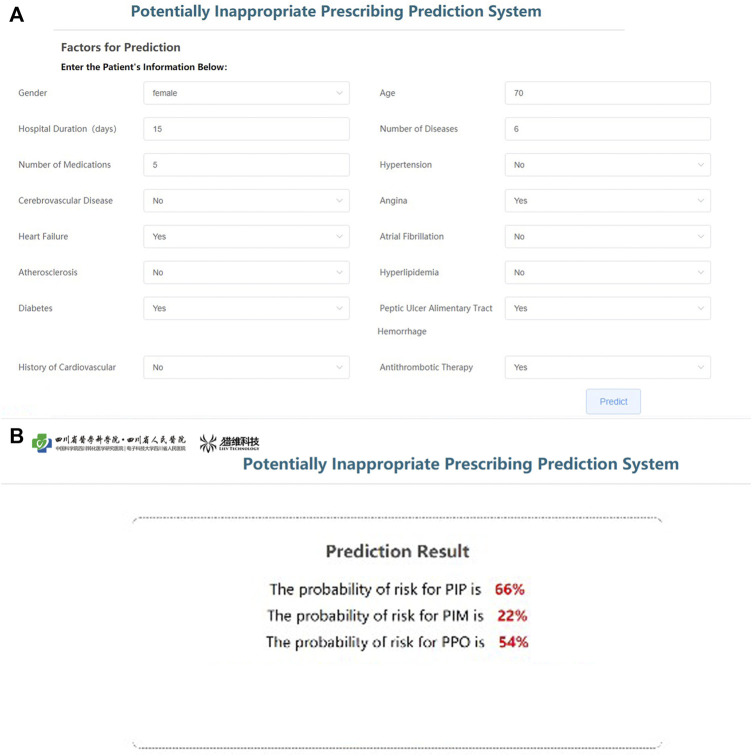
Operation interface of PIP warning platform. **(A)** User input interfaces. **(B)** User output interfaces.

Based on the parameters of the best models of PIP, PPO, and PIM, the prediction platform was established for individualized intervention. The input interface will be used to receive information on key variables in each patient (Figure 5A), and the output interface will show the risk rate of PIP, PIM, and PPO (Figure 5B). The software has obtained the Computer Software Copyright Registration Certificate (No. 7960815) received from the National Copyright Administration of the PRC (Supplementary Figure S4).

## 4 Discussion

In this study, a total of 404 elderly patients with cardiovascular disease who were hospitalized for 3–60 days were included. Five data sampling methods and three feature screening methods were used to construct 15 datasets, and 270 machine learning models were developed using 18 machine learning algorithms. AUC, accuracy, precision, recall, F1 Score, and AUPRC were used to evaluate the performance of the models. The PIP prediction platform was developed based on the parameters in the best model (the AUCs of the PIP, PPO, and PIM models were 0.8341, 0.7007 and 0.7061, respectively).

One study reported that length of stay, comorbidities, and age were associated with PIP in elderly patients (Abegaz et al., 2018). Muhlack et al. (2018) found that elderly patients with multiple diseases, frailty, and cognitive impairment were more likely to have PIM. Meanwhile, the study showed that elderly patients with lower levels of education, those taking multiple medications, and unplanned hospitalization were more likely to have PIM. Previous research suggested that the number of medications prescribed was associated with the occurrence of PIM (Nieves-Pérez et al., 2018; Ma et al., 2019). Maaroufi et al. (2021) found significant correlations between PIM and the number of medications used (at home), gender, unauthorized medications, and the number and type of comorbidities, with information on the number of medications used. Multiple results showed that comorbidities and the number of medications were key risk factors for developing PIP in the elderly. Moreover, a recent study showed that the prevalence of PIP was related to the days of hospitalization (Xu et al., 2020). According to the electronic medical record in the hospital, patients whose duration of hospitalization was between 3 and 60 days were included in the study. Patients whose length of stay was less than 3 days might die following hospitalization or have a few examinations after hospitalization and should be excluded. In this study, we found that angina, atherosclerosis, heart failure, diabetes, and the number of medications used were more strictly associated with the development of PIP in elderly patients with cardiovascular disease. These results suggest that patients with the above variables need additional care and attention. Furthermore, using these variables in similar studies may be interesting in the future.

Similar to this study, Patel et al. (2020) built prediction models using cross-validated logistic regression (CVLR) and XGBoost to screen predictors of potentially inappropriate osteoarthritis in the elderly with NSAIDs. The machine learning algorithms used in this study included two machine learning algorithms used by Patel et al. (2020). Compared to this study, Patel et al. (2020) reported a better predictive performance with an AUC of 0.8341 and an accuracy of 0.7160. However, Patel’s study did not perform external validation and had poor generalization ability. In this study, external validation was performed. Additionally, the ensemble algorithms summarized the output of the five best models (assessed by AUC) among the trained models and generated output according to the voting principle, which could help to improve the prediction performance of models. The results suggested that the model in the present study had a stronger generalization ability and higher prediction accuracy.

## 5 Limitations

This study had a number of limitations. First, this study was based on data from a single medical center in China. We are not certain if our results can also be generalized to other hospitals with a large elderly population. Second, according to the sample size results of this study and the results of other studies (Black et al., 2018; Rose et al., 2018; Hyttinen et al., 2019a; Hyttinen et al., 2019b), a larger sample size is needed to further optimize the model in the future. Third, this study was a retrospective analysis, so there were cases of incomplete data or missing records. For example, educational status has previously been demonstrated to be associated with the development of PIP. However, we lacked such data.

## 6 Conclusion

In summary, we developed a risk warning platform for potentially inappropriate prescriptions in elderly patients with cardiovascular disease who are over 65 years of age and with hospitalization between 3 and 60 days. We explored various combinations of different sampling methods, feature selection methods, and algorithms. Additionally, the contribution of variables was demonstrated by several methods. The risk warning platform could conveniently inform clinicians about the risk of PIP, which is key to the development of effective and personalized treatment strategies.

## Data Availability

The original contributions presented in the study are included in the article Supplementary Material; further inquiries can be directed to the corresponding authors.

## Supplementary material

Supplementary Figure S1 Results of feature screening in different models. **(A)** In the PIP model. **(B)** In the PPO model. **(C)** In the PIM model. (The importance of each variable was summarized and shown with the mean of the 15 datasets. The variable names are shown in Supplementary Table S1).

Supplementary Figure S2 Contribution of each variable to prediction performance in different models. **(A)** In the PIP model. **(B)** In the PPO model. **(C)** In the PIM model. (The importance of each variable was summarized when the variable was included in the models. The variable names are shown in Supplementary Table S1).

Supplementary Figure S3 Results of sample size validation **(A)** In the PIP model. **(B)** In the PPO model. **(C)** In the PIM model.

Supplementary Figure S4 Computer Software Copyright Registration Certificate.
