# Supplementary figures and images for: A machine learning-based risk warning platform for potentially inappropriate prescriptions for elderly patients with cardiovascular disease
Source: Front Pharmacol. 2022 Aug 11;13:804566. doi: 10.3389/fphar.2022.804566 (PMC9402906; doi:10.3389/fphar.2022.804566)

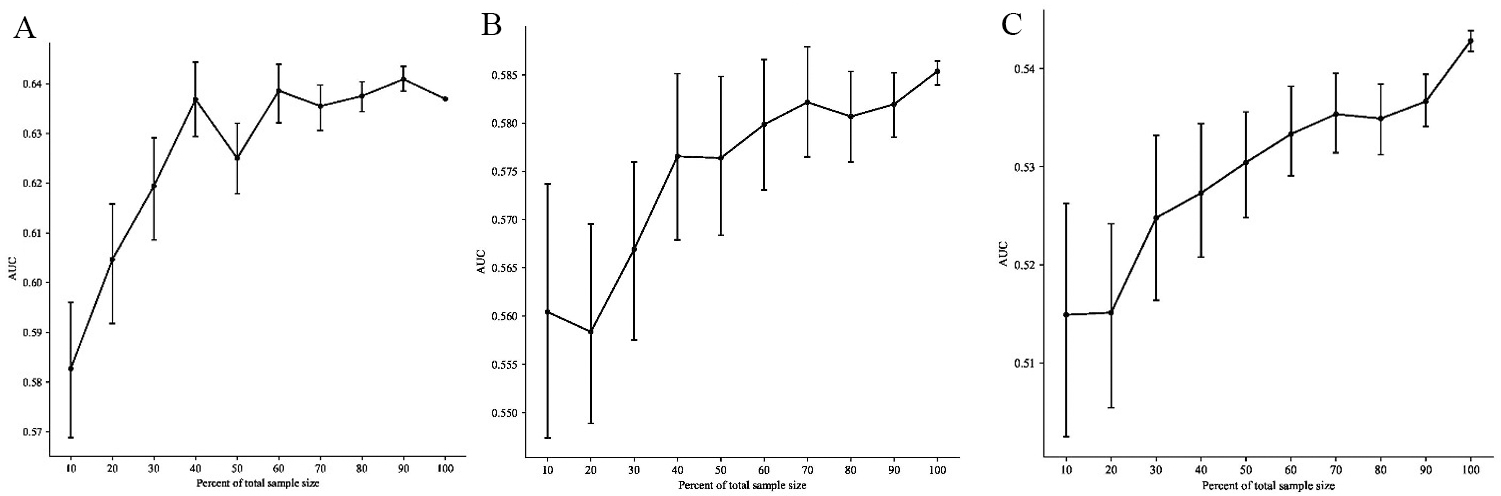

Supplement: Supplementary file 1 [file Image3.JPEG]

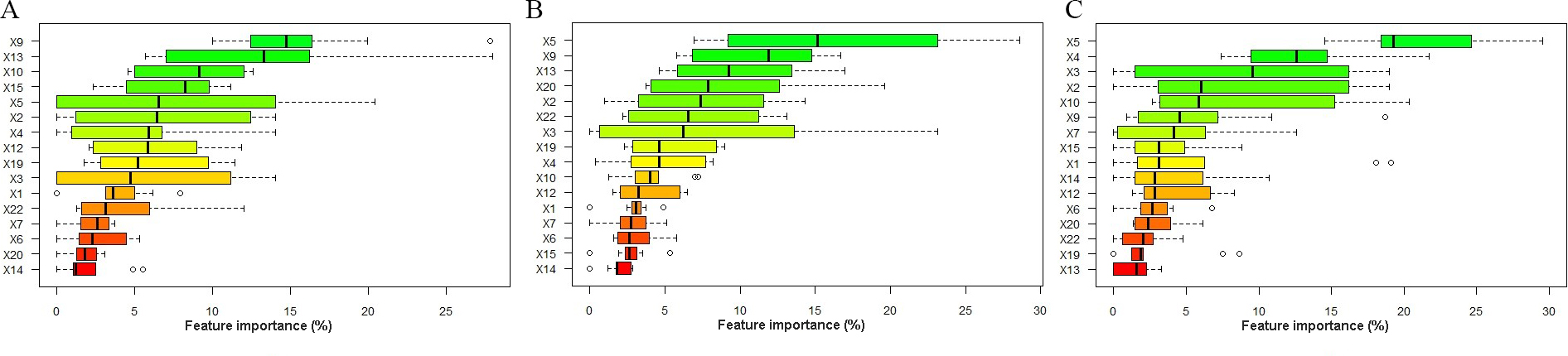

Supplement: Supplementary file 3 [file Image1.JPEG]

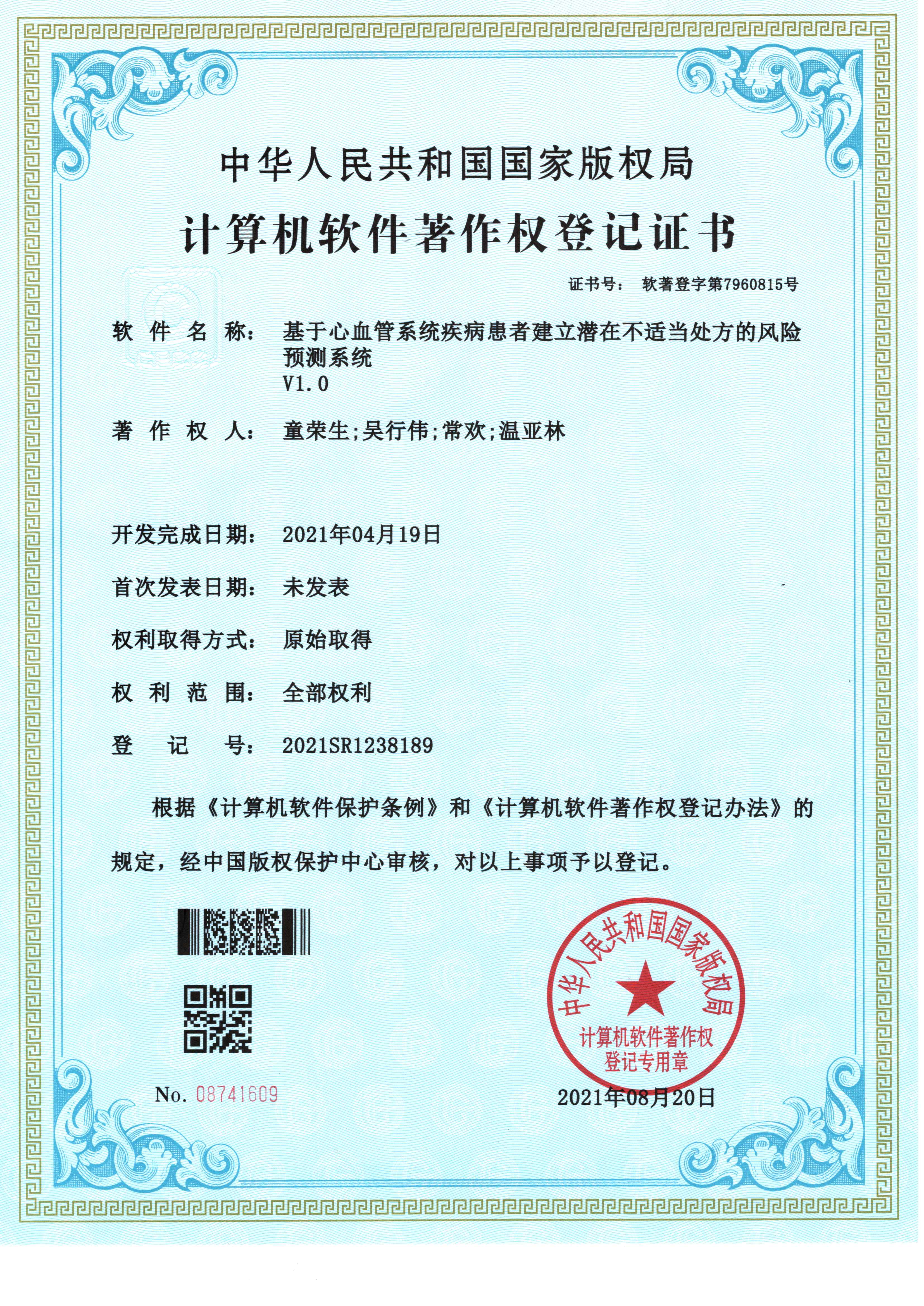

Supplement: Supplementary file 4 [file Image4.JPEG]

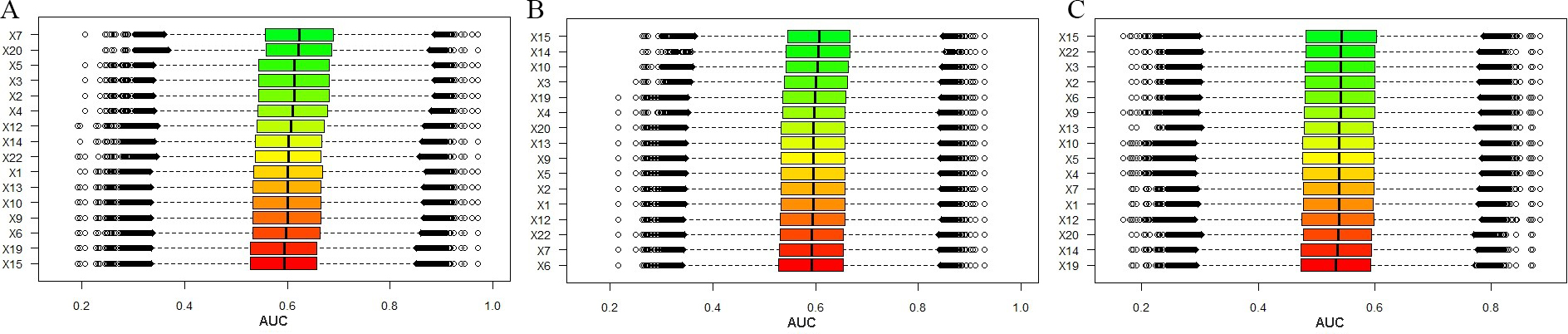

Supplement: Supplementary file 5 [file Image2.JPEG]
