# Supplementary material for: A machine learning-based risk warning platform for potentially inappropriate prescriptions for elderly patients with cardiovascular disease
Source: Front Pharmacol. 2022 Aug 11;13:804566. doi: 10.3389/fphar.2022.804566 (PMC9402906; doi:10.3389/fphar.2022.804566)
Supplement: Supplementary file 2 [file Table1.DOCX]

Supplementary Table 1 The results of internal and external validation in the PIP model

| **Internal /external validation** | **Methods** | **AUC** | | **Accuracy** | | **Precision** | | **Recall** | | **F1 Score** | |
| --- | --- | --- | --- | --- | --- | --- | --- | --- | --- | --- | --- |
|  |  | Mean±SD | 95%CI | Mean±SD | 95%CI | Mean±SD | 95%CI | Mean±SD | 95%CI | Mean±SD | 95%CI |
| **Internal validation** |  |  |  |  |  |  |  |  |  |  |  |
|  | **Data Sampling** |  |  |  |  |  |  |  |  |  |  |
|  | Borderline SMOTE | 0.878±0.097 | 0.870-0.887 | 0.808±0.097 | 0.800-0.816 | 0.847±0.134 | 0.835-0.858 | 0.782±0.086 | 0.775-0.790 | 0.807±0.087 | 0.799-0.814 |
|  | Not sampling | 0.699±0.098 | 0.690-0.707 | 0.769±0.052 | 0.764-0.773 | 0.811±0.034 | 0.808-0.814 | **0.924±0.078** | 0.917-0.931 | **0.861±0.039** | 0.858-0.865 |
|  | Random Over Sampler | 0.814±0.108 | 0.805-0.824 | 0.754±0.109 | 0.744-0.763 | 0.799±0.133 | 0.788-0.811 | 0.692±0.120 | 0.682-0.703 | 0.737±0.114 | 0.727-0.747 |
|  | Random Under Sampler | 0.730±0.137 | 0.718-0.741 | 0.679±0.120 | 0.668-0.689 | 0.706±0.151 | 0.693-0.719 | 0.661±0.172 | 0.646-0.676 | 0.669±0.128 | 0.657-0.680 |
|  | SMOTE | **0.880±0.095** | 0.872-0.889 | **0.814±0.095** | 0.806-0.823 | **0.861±0.136** | 0.849-0.872 | 0.781±0.081 | 0.774-0.788 | 0.812±0.084 | 0.805-0.819 |
|  | P value | **P<0.0001** | | **P<0.0001** | | **P<0.0001** | | **P<0.0001** | | **P<0.0001** | |
|  | **Feature Screening** |  |  |  |  |  |  |  |  |  |  |
|  | Boruta | 0.802±0.132 | 0.793-0.810 | 0.768±0.106 | 0.761-0.775 | **0.811±0.132** | 0.802-0.820 | 0.766±0.146 | 0.756-0.776 | 0.779±0.116 | 0.771-0.787 |
|  | Lasso | 0.794±0.125 | 0.786-0.802 | 0.755±0.105 | 0.748-0.762 | 0.794±0.135 | 0.785-0.803 | 0.761±0.145 | 0.751-0.771 | 0.768±0.113 | 0.760-0.775 |
|  | Not screening | 0.805±0.137 | 0.796-0.814 | **0.771±0.114** | 0.763-0.779 | 0.809±0.140 | 0.799-0.818 | **0.777±0.145** | 0.768-0.787 | **0.785±0.121** | 0.776-0.793 |
|  | P value | P=0.0793 | | **P=0.0019** | | **P=0.0218** | | **P=0.0157** | | **P=0.0008** | |
|  | **Algorithms** |  |  |  |  |  |  |  |  |  |  |
|  | AdaBoost | 0.796±0.118 | 0.777-0.815 | 0.753±0.112 | 0.735-0.771 | 0.783±0.143 | 0.760-0.806 | 0.760±0.163 | 0.733-0.786 | 0.763±0.135 | 0.741-0.785 |
|  | Bagging | 0.839±0.137 | 0.817-0.861 | 0.795±0.118 | 0.776-0.814 | 0.829±0.143 | 0.806-0.852 | 0.807±0.129 | 0.786-0.828 | 0.811±0.116 | 0.793-0.830 |
|  | Bernoulli Naïve Bayes | 0.787±0.122 | 0.767-0.806 | 0.747±0.105 | 0.730-0.764 | 0.773±0.129 | 0.752-0.794 | 0.763±0.144 | 0.740-0.786 | 0.761±0.119 | 0.742-0.781 |
|  | Decision Tree | 0.773±0.115 | 0.754-0.791 | 0.755±0.092 | 0.740-0.770 | 0.815±0.131 | 0.794-0.836 | 0.728±0.153 | 0.704-0.753 | 0.758±0.109 | 0.740-0.775 |
|  | Extra Tree | 0.815±0.112 | 0.797-0.834 | 0.785±0.084 | 0.771-0.798 | 0.839±0.114 | 0.820-0.857 | 0.769±0.138 | 0.746-0.791 | 0.792±0.094 | 0.777-0.807 |
|  | Gaussian Naïve Bayes | 0.778±0.121 | 0.759-0.798 | 0.729±0.099 | 0.713-0.746 | 0.780±0.133 | 0.758-0.801 | 0.723±0.111 | 0.705-0.741 | 0.744±0.103 | 0.727-0.761 |
|  | Gradient Boosting | 0.829±0.115 | 0.810-0.847 | 0.774±0.104 | 0.757-0.791 | 0.820±0.132 | 0.799-0.842 | 0.768±0.162 | 0.742-0.794 | 0.782±0.121 | 0.762-0.801 |
|  | KNN | 0.822±0.121 | 0.803-0.842 | 0.792±0.103 | 0.776-0.809 | **0.848±0.136** | 0.826-0.870 | 0.777±0.151 | 0.753-0.802 | 0.800±0.113 | 0.781-0.818 |
|  | LDA | 0.797±0.116 | 0.778-0.816 | 0.762±0.095 | 0.747-0.778 | 0.801±0.125 | 0.781-0.821 | 0.762±0.142 | 0.740-0.785 | 0.773±0.108 | 0.756-0.791 |
|  | Logistic Regression | 0.794±0.123 | 0.774-0.814 | 0.766±0.104 | 0.749-0.783 | 0.794±0.129 | 0.773-0.815 | 0.782±0.144 | 0.758-0.805 | 0.780±0.112 | 0.762-0.798 |
|  | Multinomial Naïve Bayes | 0.733±0.119 | 0.714-0.752 | 0.734±0.091 | 0.719-0.749 | 0.761±0.118 | 0.742-0.780 | 0.749±0.161 | 0.723-0.775 | 0.745±0.111 | 0.727-0.763 |
|  | Passive Aggressive | 0.701±0.168 | 0.674-0.728 | 0.670±0.126 | 0.650-0.691 | 0.712±0.149 | 0.688-0.736 | 0.695±0.149 | 0.671-0.719 | 0.693±0.125 | 0.673-0.714 |
|  | QDA | 0.815±0.114 | 0.796-0.833 | 0.775±0.092 | 0.760-0.789 | 0.817±0.120 | 0.798-0.837 | 0.772±0.110 | 0.755-0.790 | 0.788±0.092 | 0.773-0.803 |
|  | Random Forest | 0.837±0.150 | 0.813-0.861 | 0.798±0.123 | 0.778-0.818 | 0.833±0.146 | 0.809-0.857 | **0.812±0.141** | 0.789-0.834 | 0.815±0.123 | 0.795-0.834 |
|  | SGD | 0.798±0.119 | 0.779-0.817 | 0.760±0.101 | 0.744-0.777 | 0.790±0.123 | 0.770-0.809 | 0.776±0.167 | 0.748-0.803 | 0.772±0.119 | 0.752-0.791 |
|  | SVM | 0.837±0.128 | 0.816-0.857 | **0.804±0.090** | 0.790-0.819 | 0.846±0.116 | 0.827-0.865 | 0.803±0.119 | 0.784-0.823 | **0.817±0.091** | 0.802-0.831 |
|  | XGBoost | **0.854±0.130** | 0.833-0.875 | 0.798±0.121 | 0.779-0.818 | 0.837±0.148 | 0.813-0.861 | 0.811±0.121 | 0.792-0.831 | 0.817±0.114 | 0.798-0.835 |
|  | P value | **P<0.0001** | | **P<0.0001** | | **P<0.0001** | | **P<0.0001** | | **P<0.0001** | |
| **External validation** |  |  |  |  |  |  |  |  |  |  |  |
|  | **Data Sampling** |  |  |  |  |  |  |  |  |  |  |
|  | Borderline SMOTE | 0.579±0.094 | 0.577-0.581 | 0.621±0.082 | 0.620-0.623 | 0.801±0.060 | 0.800-0.802 | 0.692±0.097 | 0.690-0.694 | 0.739±0.070 | 0.738-0.740 |
|  | Not | 0.603±0.095 | 0.601-0.604 | **0.756±0.071** | 0.755-0.757 | 0.811±0.051 | 0.810-0.812 | **0.903±0.092** | 0.901-0.904 | **0.851±0.054** | 0.850-0.852 |
|  | Random Over Sampler | **0.643±0.101** | 0.641-0.645 | 0.616±0.100 | 0.614-0.618 | **0.853±0.065** | 0.852-0.854 | 0.623±0.143 | 0.621-0.626 | 0.710±0.096 | 0.708-0.712 |
|  | Random Under Sampler | 0.603±0.088 | 0.601-0.605 | 0.527±0.068 | 0.526-0.528 | 0.834±0.068 | 0.833-0.836 | 0.502±0.086 | 0.500-0.504 | 0.622±0.073 | 0.621-0.624 |
|  | SMOTE | 0.568±0.097 | 0.566-0.570 | 0.618±0.084 | 0.616-0.620 | 0.800±0.060 | 0.799-0.801 | 0.687±0.102 | 0.685-0.689 | 0.735±0.073 | 0.734-0.737 |
|  | P value | **P<0.0001** | | **P<0.0001** | | **P<0.0001** | | **P<0.0001** | | **P<0.0001** | |
|  | **Feature Screening** |  |  |  |  |  |  |  |  |  |  |
|  | Boruta | 0.601±0.104 | 0.600-0.603 | 0.630±0.115 | 0.628-0.632 | 0.821±0.069 | 0.820-0.822 | 0.685±0.177 | 0.682-0.687 | 0.733±0.109 | 0.731-0.734 |
|  | Lasso | 0.574±0.090 | 0.573-0.576 | 0.606±0.104 | 0.604-0.607 | 0.813±0.064 | 0.812-0.814 | 0.654±0.162 | 0.652-0.657 | 0.713±0.101 | 0.711-0.714 |
|  | Not | **0.622±0.095** | 0.620-0.623 | **0.647±0.106** | 0.646-0.649 | **0.825±0.060** | 0.824-0.826 | **0.705±0.160** | 0.703-0.708 | **0.749±0.099** | 0.748-0.751 |
|  | P value | **P<0.0001** | | **P<0.0001** | | **P<0.0001** | | **P<0.0001** | | **P<0.0001** | |
|  | **Algorithms** |  |  |  |  |  |  |  |  |  |  |
|  | AdaBoost | 0.595±0.088 | 0.592-0.598 | 0.618±0.114 | 0.614-0.622 | 0.816±0.062 | 0.813-0.818 | 0.672±0.183 | 0.666-0.679 | 0.722±0.113 | 0.718-0.726 |
|  | Bagging | 0.591±0.100 | 0.587-0.595 | 0.657±0.099 | 0.653-0.660 | 0.813±0.057 | 0.811-0.815 | 0.737±0.150 | 0.732-0.743 | 0.763±0.092 | 0.760-0.767 |
|  | Bernoulli Naive Bayes | 0.603±0.086 | 0.600-0.606 | 0.608±0.102 | 0.604-0.611 | 0.810±0.060 | 0.808-0.812 | 0.660±0.153 | 0.654-0.665 | 0.717±0.096 | 0.714-0.720 |
|  | Decision Tree | 0.553±0.105 | 0.549-0.556 | 0.600±0.109 | 0.596-0.604 | 0.816±0.080 | 0.813-0.819 | 0.643±0.162 | 0.638-0.649 | 0.707±0.102 | 0.703-0.711 |
|  | Ensemble Learning | **0.696±0.095** | 0.693-0.700 | 0.682±0.107 | 0.678-0.686 | **0.854±0.062** | 0.852-0.856 | 0.724±0.156 | 0.719-0.730 | 0.773±0.098 | 0.770-0.777 |
|  | Extra Tree | 0.591±0.090 | 0.588-0.594 | 0.625±0.112 | 0.621-0.629 | 0.822±0.066 | 0.819-0.824 | 0.675±0.172 | 0.669-0.682 | 0.728±0.105 | 0.724-0.732 |
|  | Gaussian Naive Bayes | 0.629±0.092 | 0.626-0.633 | 0.584±0.073 | 0.581-0.586 | 0.825±0.065 | 0.822-0.827 | 0.604±0.107 | 0.600-0.608 | 0.690±0.074 | 0.688-0.693 |
|  | Gradient Boosting | 0.608±0.091 | 0.605-0.611 | 0.643±0.121 | 0.638-0.647 | 0.827±0.066 | 0.825-0.830 | 0.700±0.200 | 0.693-0.708 | 0.739±0.127 | 0.734-0.743 |
|  | KNN | 0.578±0.092 | 0.575-0.581 | 0.634±0.121 | 0.630-0.638 | 0.816±0.068 | 0.814-0.819 | 0.695±0.177 | 0.688-0.701 | 0.737±0.113 | 0.733-0.741 |
|  | LDA | 0.594±0.082 | 0.591-0.597 | 0.607±0.104 | 0.603-0.610 | 0.809±0.060 | 0.807-0.811 | 0.660±0.160 | 0.654-0.665 | 0.715±0.099 | 0.712-0.719 |
|  | Logistic Regression | 0.591±0.084 | 0.588-0.594 | 0.625±0.098 | 0.621-0.628 | 0.808±0.061 | 0.806-0.810 | 0.694±0.156 | 0.688-0.700 | 0.736±0.090 | 0.733-0.739 |
|  | Multinomial Naive Bayes | 0.563±0.082 | 0.560-0.566 | 0.597±0.116 | 0.593-0.601 | 0.815±0.067 | 0.812-0.817 | 0.642±0.195 | 0.635-0.649 | 0.701±0.110 | 0.697-0.705 |
|  | Passive Aggressive | 0.578±0.102 | 0.574-0.581 | 0.579±0.081 | 0.576-0.582 | 0.822±0.068 | 0.820-0.825 | 0.598±0.114 | 0.594-0.602 | 0.685±0.085 | 0.682-0.688 |
|  | QDA | 0.608±0.089 | 0.605-0.611 | 0.618±0.096 | 0.615-0.622 | 0.817±0.060 | 0.815-0.819 | 0.667±0.139 | 0.662-0.672 | 0.726±0.090 | 0.723-0.729 |
|  | Random Forest | 0.626±0.106 | 0.622-0.629 | 0.682±0.108 | 0.678-0.686 | 0.831±0.056 | 0.829-0.833 | 0.754±0.167 | 0.748-0.760 | 0.778±0.101 | 0.775-0.782 |
|  | SGD | 0.571±0.090 | 0.568-0.574 | 0.610±0.112 | 0.606-0.614 | 0.808±0.069 | 0.806-0.811 | 0.672±0.189 | 0.666-0.679 | 0.717±0.109 | 0.714-0.721 |
|  | SVM | 0.591±0.104 | 0.587-0.595 | 0.643±0.102 | 0.640-0.647 | 0.820±0.056 | 0.818-0.822 | 0.706±0.155 | 0.700-0.711 | 0.747±0.100 | 0.744-0.751 |
|  | XGBoost | 0.620±0.101 | 0.616-0.624 | **0.687±0.105** | 0.684-0.691 | 0.829±0.057 | 0.827-0.831 | **0.762±0.146** | 0.757-0.767 | **0.786±0.093** | 0.782-0.789 |
|  | P value | **P<0.0001** | | **P<0.0001** | | **P<0.0001** | | **P<0.0001** | | **P<0.0001** | |
