# Supplementary material for: A machine learning-based risk warning platform for potentially inappropriate prescriptions for elderly patients with cardiovascular disease
Source: Front Pharmacol. 2022 Aug 11;13:804566. doi: 10.3389/fphar.2022.804566 (PMC9402906; doi:10.3389/fphar.2022.804566)
Supplement: Supplementary file 6 [file Table2.DOCX]

Supplementary Table 2 The results of internal and external validation in the PPO model

| **Internal /external validation** | **Methods** | **AUC** | | **Accuracy** | | **Precision** | | **Recall** | | **F1 Score** | |
| --- | --- | --- | --- | --- | --- | --- | --- | --- | --- | --- | --- |
|  |  | Mean±SD | 95%CI | Mean±SD | 95%CI | Mean±SD | 95%CI | Mean±SD | 95%CI | Mean±SD | 95%CI |
| **Internal validation** |  |  |  |  |  |  |  |  |  |  |  |
|  | **Data Sampling** |  |  |  |  |  |  |  |  |  |  |
|  | Borderline SMOTE | 0.878±0.097 | 0.870-0.887 | 0.808±0.097 | 0.800-0.816 | 0.847±0.134 | 0.835-0.858 | 0.782±0.086 | 0.775-0.790 | 0.807±0.087 | 0.799-0.814 |
|  | Not sampling | 0.699±0.098 | 0.690-0.707 | 0.769±0.052 | 0.764-0.773 | 0.811±0.034 | 0.808-0.814 | **0.924±0.078** | 0.917-0.931 | **0.861±0.039** | 0.858-0.865 |
|  | Random Over Sampler | 0.814±0.108 | 0.805-0.824 | 0.754±0.109 | 0.744-0.763 | 0.799±0.133 | 0.788-0.811 | 0.692±0.120 | 0.682-0.703 | 0.737±0.114 | 0.727-0.747 |
|  | Random Under Sampler | 0.730±0.137 | 0.718-0.741 | 0.679±0.120 | 0.668-0.689 | 0.706±0.151 | 0.693-0.719 | 0.661±0.172 | 0.646-0.676 | 0.669±0.128 | 0.657-0.680 |
|  | SMOTE | **0.880±0.095** | 0.872-0.889 | **0.814±0.095** | 0.806-0.823 | **0.861±0.136** | 0.849-0.872 | 0.781±0.081 | 0.774-0.788 | 0.812±0.084 | 0.805-0.819 |
|  | P value | **P<0.0001** | | **P<0.0001** | | **P<0.0001** | | **P<0.0001** | | **P<0.0001** | |
|  | **Feature Screening** |  |  |  |  |  |  |  |  |  |  |
|  | Boruta | 0.802±0.132 | 0.793-0.810 | 0.768±0.106 | 0.761-0.775 | **0.811±0.132** | 0.802-0.820 | 0.766±0.146 | 0.756-0.776 | 0.779±0.116 | 0.771-0.787 |
|  | Lasso | 0.794±0.125 | 0.786-0.802 | 0.755±0.105 | 0.748-0.762 | 0.794±0.135 | 0.785-0.803 | 0.761±0.145 | 0.751-0.771 | 0.768±0.113 | 0.760-0.775 |
|  | Not screening | 0.805±0.137 | 0.796-0.814 | **0.771±0.114** | 0.763-0.779 | 0.809±0.140 | 0.799-0.818 | **0.777±0.145** | 0.768-0.787 | **0.785±0.121** | 0.776-0.793 |
|  | P value | P=0.0793 | | **P=0.0019** | | **P=0.0218** | | **P=0.0157** | | **P=0.0008** | |
|  | **Algorithms** |  |  |  |  |  |  |  |  |  |  |
|  | AdaBoost | 0.796±0.118 | 0.777-0.815 | 0.753±0.112 | 0.735-0.771 | 0.783±0.143 | 0.760-0.806 | 0.760±0.163 | 0.733-0.786 | 0.763±0.135 | 0.741-0.785 |
|  | Bagging | 0.839±0.137 | 0.817-0.861 | 0.795±0.118 | 0.776-0.814 | 0.829±0.143 | 0.806-0.852 | 0.807±0.129 | 0.786-0.828 | 0.811±0.116 | 0.793-0.830 |
|  | Bernoulli Naïve Bayes | 0.787±0.122 | 0.767-0.806 | 0.747±0.105 | 0.730-0.764 | 0.773±0.129 | 0.752-0.794 | 0.763±0.144 | 0.740-0.786 | 0.761±0.119 | 0.742-0.781 |
|  | Decision Tree | 0.773±0.115 | 0.754-0.791 | 0.755±0.092 | 0.740-0.770 | 0.815±0.131 | 0.794-0.836 | 0.728±0.153 | 0.704-0.753 | 0.758±0.109 | 0.740-0.775 |
|  | Extra Tree | 0.815±0.112 | 0.797-0.834 | 0.785±0.084 | 0.771-0.798 | 0.839±0.114 | 0.820-0.857 | 0.769±0.138 | 0.746-0.791 | 0.792±0.094 | 0.777-0.807 |
|  | Gaussian Naïve Bayes | 0.778±0.121 | 0.759-0.798 | 0.729±0.099 | 0.713-0.746 | 0.780±0.133 | 0.758-0.801 | 0.723±0.111 | 0.705-0.741 | 0.744±0.103 | 0.727-0.761 |
|  | Gradient Boosting | 0.829±0.115 | 0.810-0.847 | 0.774±0.104 | 0.757-0.791 | 0.820±0.132 | 0.799-0.842 | 0.768±0.162 | 0.742-0.794 | 0.782±0.121 | 0.762-0.801 |
|  | KNN | 0.822±0.121 | 0.803-0.842 | 0.792±0.103 | 0.776-0.809 | **0.848±0.136** | 0.826-0.870 | 0.777±0.151 | 0.753-0.802 | 0.800±0.113 | 0.781-0.818 |
|  | LDA | 0.797±0.116 | 0.778-0.816 | 0.762±0.095 | 0.747-0.778 | 0.801±0.125 | 0.781-0.821 | 0.762±0.142 | 0.740-0.785 | 0.773±0.108 | 0.756-0.791 |
|  | Logistic Regression | 0.794±0.123 | 0.774-0.814 | 0.766±0.104 | 0.749-0.783 | 0.794±0.129 | 0.773-0.815 | 0.782±0.144 | 0.758-0.805 | 0.780±0.112 | 0.762-0.798 |
|  | Multinomial Naïve Bayes | 0.733±0.119 | 0.714-0.752 | 0.734±0.091 | 0.719-0.749 | 0.761±0.118 | 0.742-0.780 | 0.749±0.161 | 0.723-0.775 | 0.745±0.111 | 0.727-0.763 |
|  | Passive Aggressive | 0.701±0.168 | 0.674-0.728 | 0.670±0.126 | 0.650-0.691 | 0.712±0.149 | 0.688-0.736 | 0.695±0.149 | 0.671-0.719 | 0.693±0.125 | 0.673-0.714 |
|  | QDA | 0.815±0.114 | 0.796-0.833 | 0.775±0.092 | 0.760-0.789 | 0.817±0.120 | 0.798-0.837 | 0.772±0.110 | 0.755-0.790 | 0.788±0.092 | 0.773-0.803 |
|  | Random Forest | 0.837±0.150 | 0.813-0.861 | 0.798±0.123 | 0.778-0.818 | 0.833±0.146 | 0.809-0.857 | **0.812±0.141** | 0.789-0.834 | 0.815±0.123 | 0.795-0.834 |
|  | SGD | 0.798±0.119 | 0.779-0.817 | 0.760±0.101 | 0.744-0.777 | 0.790±0.123 | 0.770-0.809 | 0.776±0.167 | 0.748-0.803 | 0.772±0.119 | 0.752-0.791 |
|  | SVM | 0.837±0.128 | 0.816-0.857 | **0.804±0.090** | 0.790-0.819 | 0.846±0.116 | 0.827-0.865 | 0.803±0.119 | 0.784-0.823 | **0.817±0.091** | 0.802-0.831 |
|  | XGBoost | **0.854±0.130** | 0.833-0.875 | 0.798±0.121 | 0.779-0.818 | 0.837±0.148 | 0.813-0.861 | 0.811±0.121 | 0.792-0.831 | 0.817±0.114 | 0.798-0.835 |
|  | P value | **P<0.0001** | | **P<0.0001** | | **P<0.0001** | | **P<0.0001** | | **P<0.0001** | |
| **External validation** |  |  |  |  |  |  |  |  |  |  |  |
|  | **Data Sampling** |  |  |  |  |  |  |  |  |  |  |
|  | Borderline SMOTE | **0.632±0.082** | 0.630-0.633 | 0.673±0.070 | 0.672-0.674 | **0.777±0.062** | 0.776-0.778 | 0.727±0.093 | 0.725-0.728 | 0.748±0.067 | 0.746-0.749 |
|  | Not | 0.580±0.083 | 0.578-0.581 | 0.662±0.066 | 0.661-0.663 | 0.706±0.060 | 0.704-0.707 | **0.865±0.089** | 0.863-0.866 | **0.774±0.055** | 0.773-0.775 |
|  | Random Over Sampler | 0.574±0.082 | 0.572-0.575 | 0.589±0.077 | 0.588-0.591 | 0.723±0.073 | 0.722-0.724 | 0.641±0.112 | 0.639-0.643 | 0.674±0.081 | 0.673-0.676 |
|  | Random Under Sampler | 0.559±0.091 | 0.557-0.561 | 0.582±0.074 | 0.581-0.584 | 0.728±0.076 | 0.726-0.729 | 0.617±0.121 | 0.615-0.620 | 0.660±0.085 | 0.659-0.662 |
|  | SMOTE | 0.629±0.083 | 0.627-0.630 | **0.674±0.071** | 0.673-0.676 | 0.767±0.065 | 0.766-0.768 | 0.745±0.086 | 0.743-0.747 | 0.753±0.064 | 0.752-0.755 |
|  | P value | **P<0.0001** | | **P<0.0001** | | **P<0.0001** | | **P<0.0001** | | **P<0.0001** | |
|  | **Feature Screening** |  |  |  |  |  |  |  |  |  |  |
|  | Boruta | 0.596±0.087 | 0.594-0.597 | 0.633±0.082 | 0.632-0.634 | 0.740±0.072 | 0.739-0.741 | 0.712±0.133 | 0.710-0.714 | 0.718±0.085 | 0.717-0.720 |
|  | Lasso | 0.584±0.091 | 0.583-0.585 | 0.629±0.085 | 0.628-0.631 | 0.734±0.074 | 0.732-0.735 | 0.716±0.137 | 0.714-0.718 | 0.717±0.086 | 0.716-0.719 |
|  | Not | **0.604±0.089** | 0.603-0.605 | **0.646±0.081** | 0.645-0.647 | **0.746±0.071** | 0.745-0.747 | **0.728±0.131** | 0.727-0.730 | **0.730±0.083** | 0.729-0.731 |
|  | P value | **P<0.0001** | | **P<0.0001** | | **P<0.0001** | | **P<0.0001** | | **P<0.0001** | |
|  | **Algorithms** |  |  |  |  |  |  |  |  |  |  |
|  | AdaBoost | 0.600±0.077 | 0.597-0.603 | 0.655±0.070 | 0.652-0.657 | 0.736±0.070 | 0.734-0.739 | **0.771±0.102** | 0.768-0.775 | 0.749±0.062 | 0.746-0.751 |
|  | Bagging | 0.617±0.079 | 0.614-0.620 | 0.635±0.076 | 0.632-0.637 | 0.743±0.073 | 0.740-0.745 | 0.709±0.102 | 0.705-0.713 | 0.721±0.071 | 0.719-0.724 |
|  | Bernoulli Naive Bayes | 0.595±0.085 | 0.592-0.598 | 0.654±0.078 | 0.651-0.657 | 0.739±0.070 | 0.737-0.742 | 0.760±0.112 | 0.756-0.764 | 0.745±0.071 | 0.742-0.747 |
|  | Decision Tree | 0.526±0.074 | 0.524-0.529 | 0.537±0.075 | 0.534-0.540 | 0.706±0.079 | 0.703-0.709 | 0.559±0.169 | 0.553-0.565 | 0.608±0.105 | 0.604-0.612 |
|  | Ensemble Learning | **0.676±0.070** | 0.674-0.679 | **0.678±0.071** | 0.676-0.681 | **0.769±0.067** | 0.766-0.771 | 0.763±0.143 | 0.758-0.768 | **0.756±0.078** | 0.753-0.759 |
|  | Extra Tree | 0.560±0.087 | 0.556-0.563 | 0.591±0.084 | 0.588-0.594 | 0.718±0.081 | 0.715-0.721 | 0.659±0.132 | 0.655-0.664 | 0.679±0.088 | 0.676-0.682 |
|  | Gaussian Naive Bayes | 0.576±0.080 | 0.573-0.579 | 0.660±0.063 | 0.658-0.662 | 0.745±0.069 | 0.742-0.747 | 0.766±0.067 | 0.763-0.768 | 0.752±0.051 | 0.751-0.754 |
|  | Gradient Boosting | 0.579±0.083 | 0.576-0.582 | 0.611±0.068 | 0.608-0.613 | 0.729±0.067 | 0.726-0.731 | 0.683±0.114 | 0.679-0.687 | 0.699±0.071 | 0.697-0.702 |
|  | KNN | 0.578±0.091 | 0.575-0.581 | 0.653±0.076 | 0.651-0.656 | 0.738±0.070 | 0.735-0.740 | 0.765±0.114 | 0.761-0.769 | 0.746±0.068 | 0.744-0.749 |
|  | LDA | 0.596±0.086 | 0.593-0.599 | 0.662±0.086 | 0.659-0.665 | 0.749±0.072 | 0.747-0.752 | 0.754±0.124 | 0.750-0.759 | 0.747±0.080 | 0.744-0.749 |
|  | Logistic Regression | 0.587±0.085 | 0.584-0.590 | 0.662±0.083 | 0.659-0.665 | 0.754±0.068 | 0.751-0.756 | 0.745±0.125 | 0.740-0.749 | 0.744±0.080 | 0.741-0.747 |
|  | Multinomial Naive Bayes | 0.617±0.082 | 0.614-0.620 | 0.624±0.083 | 0.621-0.627 | 0.733±0.073 | 0.730-0.736 | 0.711±0.172 | 0.705-0.717 | 0.710±0.091 | 0.706-0.713 |
|  | Passive Aggressive | 0.595±0.098 | 0.591-0.599 | 0.604±0.089 | 0.601-0.607 | 0.734±0.079 | 0.731-0.736 | 0.659±0.143 | 0.654-0.664 | 0.685±0.095 | 0.682-0.689 |
|  | QDA | 0.589±0.085 | 0.586-0.592 | 0.649±0.071 | 0.647-0.652 | 0.740±0.066 | 0.737-0.742 | 0.746±0.108 | 0.742-0.749 | 0.738±0.068 | 0.736-0.741 |
|  | Random Forest | 0.616±0.092 | 0.612-0.619 | 0.640±0.074 | 0.637-0.643 | 0.744±0.075 | 0.741-0.747 | 0.724±0.119 | 0.720-0.728 | 0.726±0.075 | 0.724-0.729 |
|  | SGD | 0.594±0.087 | 0.591-0.597 | 0.641±0.079 | 0.639-0.644 | 0.747±0.071 | 0.744-0.749 | 0.719±0.130 | 0.714-0.724 | 0.726±0.079 | 0.723-0.729 |
|  | SVM | 0.583±0.089 | 0.579-0.586 | 0.647±0.073 | 0.644-0.649 | 0.742±0.066 | 0.740-0.744 | 0.738±0.114 | 0.734-0.742 | 0.735±0.069 | 0.732-0.737 |
|  | XGBoost | 0.621±0.086 | 0.618-0.624 | 0.647±0.070 | 0.644-0.649 | 0.755±0.067 | 0.753-0.758 | 0.709±0.091 | 0.706-0.713 | 0.728±0.066 | 0.726-0.731 |
|  | P value | **P<0.0001** | | **P<0.0001** | | **P<0.0001** | | **P<0.0001** | | **P<0.0001** | |
