# Supplementary material for: A machine learning-based risk warning platform for potentially inappropriate prescriptions for elderly patients with cardiovascular disease
Source: Front Pharmacol. 2022 Aug 11;13:804566. doi: 10.3389/fphar.2022.804566 (PMC9402906; doi:10.3389/fphar.2022.804566)
Supplement: Supplementary file 7 [file Table3.DOCX]

Supplementary Table 3 The results of internal and external validation in the PIM model

| **Internal /external validation** | **Methods** | **AUC** | | **Accuracy** | | **Precision** | | **Recall** | | **F1 Score** | |
| --- | --- | --- | --- | --- | --- | --- | --- | --- | --- | --- | --- |
|  |  | Mean±SD | 95%CI | Mean±SD | 95%CI | Mean±SD | 95%CI | Mean±SD | 95%CI | Mean±SD | 95%CI |
| **Internal validation** |  |  |  |  |  |  |  |  |  |  |  |
|  | **Data Sampling** |  |  |  |  |  |  |  |  |  |  |
|  | Borderline SMOTE | 0.736±0.124 | 0.725-0.746 | 0.685±0.103 | 0.676-0.694 | 0.670±0.096 | 0.661-0.678 | 0.735±0.186 | 0.718-0.751 | 0.691±0.123 | 0.681-0.702 |
|  | Not sampling | 0.665±0.088 | 0.657-0.672 | 0.704±0.055 | 0.699-0.709 | 0.378±0.262 | 0.356-0.401 | 0.203±0.170 | 0.188-0.218 | 0.245±0.175 | 0.230-0.260 |
|  | Random Over Sampler | **0.786±0.126** | 0.775-0.797 | **0.726±0.115** | 0.716-0.736 | **0.713±0.102** | 0.704-0.721 | **0.756±0.163** | 0.741-0.770 | **0.729±0.123** | 0.718-0.740 |
|  | Random Under Sampler | 0.693±0.106 | 0.684-0.702 | 0.637±0.098 | 0.628-0.645 | 0.652±0.118 | 0.641-0.662 | 0.628±0.151 | 0.615-0.641 | 0.629±0.108 | 0.619-0.638 |
|  | SMOTE | 0.750±0.129 | 0.739-0.761 | 0.696±0.114 | 0.686-0.706 | 0.683±0.106 | 0.674-0.693 | 0.730±0.191 | 0.713-0.746 | 0.697±0.132 | 0.686-0.708 |
|  | P value | **P<0.0001** | | **P<0.0001** | | **P<0.0001** | | **P<0.0001** | | **P<0.0001** | |
|  | **Feature Screening** |  |  |  |  |  |  |  |  |  |  |
|  | Boruta | **0.728±0.125** | 0.719-0.736 | **0.692±0.105** | 0.684-0.699 | **0.625±0.179** | 0.613-0.637 | **0.613±0.264** | 0.595-0.631 | **0.604±0.216** | 0.589-0.619 |
|  | Lasso | 0.724±0.119 | 0.716-0.732 | 0.687±0.100 | 0.680-0.694 | 0.618±0.196 | 0.605-0.631 | 0.610±0.270 | 0.592-0.628 | 0.597±0.222 | 0.582-0.612 |
|  | Not screening | 0.725±0.126 | 0.717-0.734 | 0.690±0.106 | 0.683-0.698 | 0.614±0.206 | 0.600-0.628 | 0.607±0.278 | 0.588-0.626 | 0.594±0.234 | 0.578-0.609 |
|  | P value | P=0.8222 | | P=0.8895 | | P=0.9602 | | P=0.9910 | | P=0.9223 | |
|  | **Algorithms** |  |  |  |  |  |  |  |  |  |  |
|  | AdaBoost | 0.732±0.092 | 0.718-0.747 | 0.689±0.078 | 0.676-0.701 | 0.643±0.146 | 0.619-0.666 | 0.599±0.255 | 0.558-0.640 | 0.597±0.198 | 0.565-0.629 |
|  | Bagging | 0.815±0.142 | 0.792-0.838 | 0.754±0.104 | 0.738-0.771 | 0.682±0.184 | 0.652-0.712 | 0.705±0.271 | 0.662-0.749 | 0.683±0.222 | 0.647-0.719 |
|  | Bernoulli Naïve Bayes | 0.675±0.101 | 0.659-0.691 | 0.643±0.095 | 0.628-0.658 | 0.597±0.137 | 0.575-0.619 | 0.561±0.236 | 0.523-0.599 | 0.557±0.179 | 0.529-0.586 |
|  | Decision Tree | 0.687±0.113 | 0.669-0.705 | 0.685±0.086 | 0.671-0.698 | 0.604±0.208 | 0.570-0.637 | 0.590±0.272 | 0.546-0.634 | 0.582±0.231 | 0.544-0.619 |
|  | Extra Tree | 0.740±0.106 | 0.723-0.757 | 0.718±0.090 | 0.704-0.733 | 0.663±0.165 | 0.637-0.690 | 0.665±0.253 | 0.625-0.706 | 0.647±0.198 | 0.615-0.679 |
|  | Gaussian Naïve Bayes | 0.671±0.094 | 0.656-0.686 | 0.638±0.085 | 0.624-0.651 | 0.579±0.132 | 0.558-0.601 | 0.594±0.229 | 0.557-0.631 | 0.572±0.164 | 0.546-0.599 |
|  | Gradient Boosting | 0.782±0.107 | 0.765-0.799 | 0.718±0.089 | 0.703-0.732 | 0.664±0.166 | 0.637-0.691 | 0.633±0.281 | 0.588-0.678 | 0.623±0.229 | 0.587-0.660 |
|  | KNN | 0.755±0.115 | 0.736-0.773 | 0.736±0.108 | 0.718-0.753 | 0.660±0.234 | 0.622-0.698 | 0.645±0.316 | 0.594-0.696 | 0.627±0.267 | 0.584-0.670 |
|  | LDA | 0.704±0.086 | 0.691-0.718 | 0.666±0.080 | 0.653-0.679 | 0.623±0.130 | 0.602-0.644 | 0.581±0.220 | 0.546-0.617 | 0.586±0.166 | 0.559-0.613 |
|  | Logistic Regression | 0.703±0.082 | 0.689-0.716 | 0.666±0.076 | 0.654-0.679 | 0.614±0.161 | 0.588-0.640 | 0.566±0.258 | 0.524-0.607 | 0.565±0.207 | 0.531-0.598 |
|  | Multinomial Naïve Bayes | 0.649±0.078 | 0.636-0.661 | 0.645±0.073 | 0.634-0.657 | 0.498±0.257 | 0.456-0.539 | 0.522±0.293 | 0.474-0.569 | 0.504±0.265 | 0.461-0.546 |
|  | Passive Aggressive | 0.592±0.120 | 0.573-0.611 | 0.575±0.095 | 0.559-0.590 | 0.513±0.149 | 0.489-0.537 | 0.526±0.206 | 0.493-0.559 | 0.509±0.162 | 0.483-0.535 |
|  | QDA | 0.731±0.090 | 0.717-0.746 | 0.684±0.081 | 0.671-0.697 | 0.631±0.134 | 0.609-0.653 | 0.612±0.241 | 0.573-0.651 | 0.605±0.182 | 0.576-0.635 |
|  | Random Forest | **0.818±0.138** | 0.796-0.840 | 0.758±0.117 | 0.739-0.777 | **0.702±0.182** | 0.672-0.731 | 0.689±0.281 | 0.644-0.734 | 0.678±0.231 | 0.641-0.716 |
|  | SGD | 0.707±0.089 | 0.693-0.722 | 0.666±0.073 | 0.654-0.678 | 0.519±0.270 | 0.475-0.563 | 0.543±0.307 | 0.494-0.593 | 0.523±0.277 | 0.479-0.568 |
|  | SVM | 0.763±0.127 | 0.742-0.783 | 0.720±0.099 | 0.704-0.736 | 0.635±0.223 | 0.599-0.671 | 0.637±0.302 | 0.588-0.685 | 0.617±0.257 | 0.575-0.658 |
|  | XGBoost | 0.815±0.130 | 0.794-0.836 | **0.760±0.114** | 0.742-0.779 | 0.700±0.176 | 0.671-0.728 | **0.705±0.264** | 0.662-0.748 | **0.692±0.213** | 0.657-0.726 |
|  | P value | **P<0.0001** | | **P<0.0001** | | **P<0.0001** | | **P<0.0001** | | **P<0.0001** | |
| **External validation** |  |  |  |  |  |  |  |  |  |  |  |
|  | **Data Sampling** |  |  |  |  |  |  |  |  |  |  |
|  | Borderline SMOTE | 0.512±0.086 | 0.510-0.513 | 0.549±0.091 | 0.548-0.551 | 0.283±0.103 | 0.281-0.285 | 0.420±0.184 | 0.417-0.424 | 0.324±0.110 | 0.322-0.326 |
|  | Not | 0.523±0.093 | 0.522-0.525 | **0.659±0.073** | 0.658-0.661 | 0.163±0.166 | 0.160-0.166 | 0.104±0.131 | 0.101-0.106 | 0.115±0.121 | 0.113-0.118 |
|  | Random Over Sampler | 0.537±0.089 | 0.535-0.538 | 0.608±0.072 | 0.607-0.610 | 0.314±0.113 | 0.311-0.316 | 0.385±0.174 | 0.382-0.388 | 0.332±0.122 | 0.330-0.335 |
|  | Random Under Sampler | **0.563±0.089** | 0.561-0.565 | 0.572±0.078 | 0.570-0.573 | 0.323±0.094 | 0.322-0.325 | **0.517±0.133** | 0.514-0.519 | **0.392±0.097** | 0.390-0.394 |
|  | SMOTE | 0.553±0.088 | 0.552-0.555 | 0.598±0.086 | 0.597-0.600 | **0.330±0.110** | 0.328-0.332 | 0.432±0.162 | 0.429-0.435 | 0.359±0.107 | 0.357-0.361 |
|  | P value | **P<0.0001** | | **P<0.0001** | | **P<0.0001** | | **P<0.0001** | | **P<0.0001** | |
|  | **Feature Screening** |  |  |  |  |  |  |  |  |  |  |
|  | Boruta | **0.546±0.089** | 0.545-0.547 | 0.596±0.087 | 0.595-0.598 | 0.286±0.131 | 0.284-0.287 | **0.383±0.218** | 0.379-0.386 | **0.310±0.148** | 0.308-0.312 |
|  | Lasso | 0.528±0.095 | 0.527-0.530 | 0.585±0.086 | 0.584-0.587 | 0.271±0.128 | 0.269-0.273 | 0.370±0.212 | 0.367-0.373 | 0.298±0.146 | 0.296-0.300 |
|  | Not | 0.539±0.087 | 0.537-0.540 | **0.610±0.091** | 0.609-0.612 | **0.291±0.144** | 0.289-0.293 | 0.362±0.205 | 0.359-0.365 | 0.305±0.151 | 0.303-0.308 |
|  | P value | **P<0.0001** | | **P<0.0001** | | **P<0.0001** | | **P<0.0001** | | **P<0.0001** | |
|  | **Algorithms** |  |  |  |  |  |  |  |  |  |  |
|  | AdaBoost | 0.517±0.080 | 0.514-0.520 | 0.578±0.068 | 0.576-0.581 | 0.253±0.135 | 0.249-0.258 | 0.408±0.223 | 0.400-0.416 | 0.307±0.163 | 0.302-0.313 |
|  | Bagging | 0.535±0.086 | 0.531-0.538 | 0.611±0.083 | 0.608-0.614 | 0.271±0.120 | 0.267-0.276 | 0.246±0.138 | 0.241-0.251 | 0.242±0.102 | 0.238-0.246 |
|  | Bernoulli Naive Bayes | 0.539±0.080 | 0.536-0.542 | 0.581±0.080 | 0.578-0.584 | 0.285±0.118 | 0.280-0.289 | 0.418±0.205 | 0.410-0.425 | 0.326±0.146 | 0.321-0.331 |
|  | Decision Tree | 0.509±0.084 | 0.506-0.512 | 0.609±0.081 | 0.606-0.611 | 0.274±0.148 | 0.269-0.280 | 0.339±0.202 | 0.332-0.347 | 0.288±0.155 | 0.283-0.294 |
|  | Ensemble Learning | **0.637±0.076** | 0.635-0.640 | 0.647±0.072 | 0.644-0.650 | **0.350±0.147** | 0.345-0.356 | 0.440±0.217 | 0.432-0.447 | **0.375±0.155** | 0.369-0.380 |
|  | Extra Tree | 0.499±0.104 | 0.496-0.503 | 0.586±0.086 | 0.583-0.589 | 0.263±0.135 | 0.258-0.268 | 0.359±0.238 | 0.351-0.368 | 0.286±0.159 | 0.280-0.292 |
|  | Gaussian Naive Bayes | 0.572±0.088 | 0.569-0.575 | 0.567±0.093 | 0.564-0.570 | 0.299±0.100 | 0.295-0.302 | 0.449±0.196 | 0.442-0.456 | 0.343±0.118 | 0.339-0.347 |
|  | Gradient Boosting | 0.484±0.084 | 0.481-0.487 | 0.617±0.079 | 0.614-0.620 | 0.295±0.135 | 0.290-0.299 | 0.333±0.196 | 0.326-0.340 | 0.295±0.149 | 0.289-0.300 |
|  | KNN | 0.549±0.090 | 0.546-0.552 | 0.622±0.081 | 0.619-0.624 | 0.265±0.153 | 0.260-0.271 | 0.322±0.210 | 0.315-0.330 | 0.280±0.165 | 0.274-0.286 |
|  | LDA | 0.551±0.077 | 0.549-0.554 | 0.586±0.083 | 0.583-0.589 | 0.313±0.103 | 0.309-0.317 | 0.432±0.167 | 0.426-0.438 | 0.349±0.115 | 0.345-0.353 |
|  | Logistic Regression | 0.559±0.077 | 0.556-0.562 | 0.587±0.078 | 0.584-0.590 | 0.300±0.108 | 0.296-0.303 | 0.424±0.196 | 0.417-0.431 | 0.335±0.134 | 0.330-0.340 |
|  | Multinomial Naive Bayes | 0.501±0.073 | 0.499-0.504 | 0.557±0.102 | 0.553-0.561 | 0.228±0.132 | 0.223-0.233 | 0.414±0.234 | 0.406-0.423 | 0.291±0.163 | 0.285-0.297 |
|  | Passive Aggressive | 0.517±0.088 | 0.513-0.520 | 0.554±0.095 | 0.551-0.557 | 0.305±0.097 | 0.302-0.309 | **0.487±0.192** | 0.480-0.494 | 0.361±0.106 | 0.358-0.365 |
|  | QDA | 0.540±0.078 | 0.537-0.543 | 0.581±0.085 | 0.578-0.584 | 0.282±0.112 | 0.278-0.286 | 0.367±0.172 | 0.360-0.373 | 0.307±0.126 | 0.303-0.312 |
|  | Random Forest | 0.518±0.087 | 0.515-0.521 | 0.617±0.086 | 0.614-0.620 | 0.264±0.146 | 0.258-0.269 | 0.237±0.158 | 0.231-0.242 | 0.234±0.133 | 0.229-0.239 |
|  | SGD | 0.550±0.078 | 0.548-0.553 | 0.578±0.101 | 0.575-0.582 | 0.245±0.143 | 0.240-0.250 | 0.432±0.244 | 0.423-0.441 | 0.309±0.174 | 0.302-0.315 |
|  | SVM | 0.564±0.094 | 0.561-0.568 | 0.626±0.079 | 0.624-0.629 | 0.260±0.166 | 0.254-0.266 | 0.308±0.211 | 0.300-0.315 | 0.272±0.171 | 0.266-0.278 |
|  | XGBoost | 0.535±0.087 | 0.532-0.538 | **0.649±0.072** | 0.646-0.651 | 0.332±0.141 | 0.327-0.337 | 0.271±0.147 | 0.265-0.276 | 0.280±0.119 | 0.276-0.284 |
|  | P value | **P<0.0001** | | **P<0.0001** | | **P<0.0001** | | **P<0.0001** | | **P<0.0001** | |
